# A new scoring system to predict the risk of late recurrence in extended follow‐up after atrial fibrillation catheter ablation: APCEL score

**DOI:** 10.1002/joa3.70048

**Published:** 2025-03-24

**Authors:** Taner Ulus, Ahmet Şekip Ahmadi, Ertuğrul Çolak

**Affiliations:** ^1^ Department of Cardiology Eskişehir Osmangazi University Eskişehir Turkey; ^2^ Department of Biostatistics Faculty of Medicine, Eskişehir Osmangazi University Eskişehir Turkey

**Keywords:** atrial fibrillation, catheter ablation, follow‐up, recurrence, scoring system

## Abstract

**Background:**

In studies where risk scores used to determine the risk of late recurrence after atrial fibrillation (AF) catheter ablation were defined, significant differences were observed in terms of parameters such as post‐procedural follow‐up time, pre‐procedural AF time, energy sources used for ablation, and cut‐off values of left atrium (LA) diameter. Considering all these factors, we aimed to develop a new recurrence risk score for prolonged follow‐up after AF ablation.

**Methods:**

The study included 206 patients who underwent index AF catheter ablation for paroxysmal or persistent AF. Independent predictors of late recurrence were identified at a median follow‐up of 40 months (range: 21–57), and a risk score was created. The predictive ability of this score for late recurrence was compared with that of other risk scores.

**Results:**

Independent predictors of late recurrence development included pre‐ablation AF duration >19 months, persistent AF, early recurrence, chronic obstructive pulmonary disease, and LA volume index >31 mL/m^2^. The APCEL risk score, derived from these factors (Early recurrence: 3 points, AF duration >19 months: 2 points, others: 1 point), demonstrated good predictive performance for late recurrence at 6th [AUC: 0.940, 95% CI: 0.896–0.983], 12th [AUC: 0.865, 95% CI: 0.796–0.932], 24th [AUC: 0.814, 95% CI: 0.743–0.885], and 36th months [AUC: 0.798, 95% CI: 0.726–0.868].

**Conclusions:**

The APCEL score, calculated at the end of the blanking period for patients who underwent AF ablation, can effectively identify those at high risk of late recurrence during extended follow‐up.

## Graphical abstract

- We aimed to create a new scoring system in extended follow‐up after atrial fibrillation (AF) ablation.
- The APCEL score (early recurrence: 3 points, AF duration >19 months: 2 points, persistent AF, chronic obstructive pulmonary disease, and left atrial volume index >31 mL/m^2^: 1 point) had a good predictive ability for late recurrence after AF ablation.

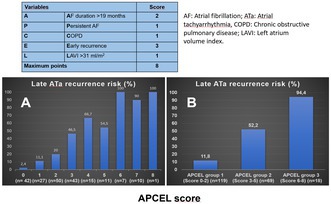

## 1 INTRODUCTION

With the growth of evidence and technological advancements, atrial fibrillation (AF) catheter ablation is increasingly performed. Current guidelines recommend early ablation for young, low‐comorbidity AF patients.^1^, ^2^ The rise in ablation procedures has led to more patients presenting with recurrent atrial tachyarrhythmias (ATa) [AF, Atrial flutter (AFL), or atrial tachycardia] after AF ablation. Some recurrences may be even more symptomatic than the pre‐ablation arrhythmia and may impair the patient's quality of life. Identifying high‐risk patients for recurrence post‐AF ablation is crucial.

Although many risk scores have been defined to determine the risk of late recurrence after AF ablation, there are important methodological differences between the studies in which such scores were developed.^3^, ^4^, ^5^, ^6^, ^7^ While the follow‐up period in some of these studies was not long enough (approximately 12 months),^3^, ^5^ some did not examine early recurrence, which is an important predictor for late recurrence.^3^, ^4^, ^5^ Some studies included patients who underwent only cryo‐balloon (CB),^5^, ^6^ while others included patients who underwent radiofrequency (RF) ablation only.^3^, ^4^, ^7^ In some studies, linear lesions were created during the first ablation procedure, which may have affected the development of ATa recurrence.^3^, ^7^ These scoring systems generally used the left atrium (LA) anteroposterior diameter. However, LA enlargement is usually asymmetrical and LA volume is a more realistic measurement of LA size.^8^ As a result, the scoring systems mentioned above have different recurrence predictors, different cut‐off values for the LA diameter,^3^, ^5^, ^6^, ^7^ and these are difficult to use routinely in daily practice.

It is indeed possible to predict the risk of late recurrence in patients undergoing catheter ablation for AF by employing various pre‐procedural risk scores.^3^, ^4^, ^5^ However, it is noteworthy that symptomatic AF patients identified as high risk for late recurrence based on these scores may still derive significant therapeutic benefits from catheter ablation. In our study, we included early recurrence in our risk assessment model, as it correlates with key factors contributing to late recurrence, such as inflammation and oxidative stress.^2^ Our aim is to create a new scoring system that considers the challenges mentioned above in extended follow‐up after AF catheter ablation, with a focus on identifying predictors of late recurrence. Thus, we aimed to use our risk score to identify individuals who would need closer monitoring and tighter risk factor control after catheter ablation.

## 2 METHODS

**FIGURE 1:**
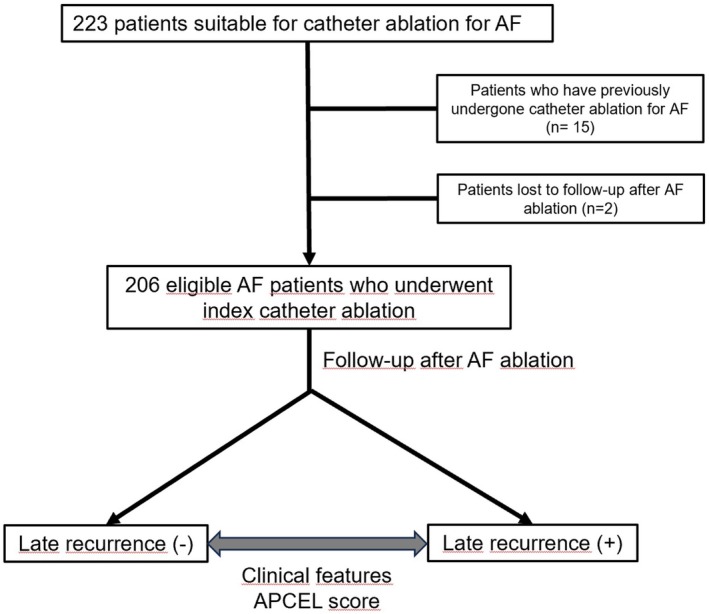
Flowchart of the study.

This retrospective study included patients who underwent index pulmonary vein (PV) isolation for paroxysmal and persistent AF between January 2017 and January 2024. The definition of paroxysmal and persistent AF was made by the guidelines.^1^ Patients who had previously undergone catheter ablation due to AF or who did not come for follow‐up were excluded from the study. Other exclusion criteria were as follows: Those with thrombi in the LA, those with an LA diameter >55 mm, those with severe heart valve disease, those who developed AF due to hyperthyroidism, those with AF in the course of acute coronary syndrome, those with a history of recent heart surgery, those with severe comorbidities, and pregnant women. As a result, 206 patients were included in the study. The flowchart of the study is given in Figure 1.

The main clinical characteristics of the patients and medications were recorded. Preoperative transthoracic echo findings were noted. The LA volume was indexed to the patient's body surface area and LA volume index (LAVI) was calculated. All echocardiographic measurements were made in accordance with the recommendations of current guidelines.^9^ Our study was carried out in accordance with the Declaration of Helsinki and local ethics committee approval was received before the study (Approval date: 05.06.2024, approval No: 35).

### 2.1 Ablation procedure

If patients were taking antiarrhythmic medication before the procedure, they were discontinued at least five half‐lives before the procedure. Amiodarone was discontinued at least 1 month before the procedure. All patients underwent contrast‐enhanced thorax computed tomography to visualize LA and PV anatomy preprocedure. All patients underwent intraoperative transesophageal echo (TEE) immediately prior to AF ablation to rule out the presence of thrombus in LA and for safe transseptal puncture. In patients who were taking an anticoagulant before the ablation, the procedure was performed without discontinuing the anticoagulant; in other cases, oral anticoagulant was started as soon as hemostasis was achieved after the procedure.

For AF ablation with CB, conscious sedation was administered, while general anesthesia was preferred for RF method procedures. A decapolar catheter was introduced into the coronary sinus (St. Jude Medical, St Paul, Minn, USA). Transseptal puncture (TSP) was performed under fluoroscopy and TEE guidance. Before TSP, 10.000 U IV heparin was administered to all patients, and additional heparin boluses were administered if necessary, keeping the activated clotting time at 300–350 s throughout the procedure. In patients with AF, sinus rhythm was attempted to be established with electrical cardioversion at the beginning of the procedure, if possible.

For CB ablation, a steerable sheath (FlexCath, Medtronic CryoCath, Minneapolis, US) was placed in the LA over the guide wire. A 28‐mm CB catheter (Medtronic, Inc., Minneapolis, MN) with an inner circular mapping catheter (Achieve Advance™ mapping catheter 20 mm, Medtronic) was sent to LA. While CB was applied to the PVs, PV isolation was monitored in real time from the circular mapping catheter. Freeze duration and bonus freeze requirements were determined by taking into account the relevant documents according to ‘time to isolation’.^10^ While freezing the right‐sided PVs, right hemidiaphragmatic contraction was manually monitored. PV entrance and exit blocks were tested immediately after freezing and after a 30‐min waiting period.

A 3‐dimensional mapping system was used for RF ablation (EnSite Precision, Abbott, or CARTO, Biosense Webster). First, LA and PV anatomies and LA voltage map were created via a multipolar mapping catheter (Advisor HD Grid, Abbott or Pentaray, Biosense WebsterBiosense Webster). Then, continuous circumferential ablation lines were created in the antrum of the PVs using an irrigated ablation catheter (TactiCath, Abbott, or SmartTouch, Biosense Webster). Finally, the multipolar mapping catheter was placed in the PVs and the entrance and exit blocks were checked.

Acute procedure success was accepted as a bidirectional block in all PVs at the end of the procedure. Class IC or class III antiarrhythmic drugs were started for 3 months after the procedure. Anticoagulant treatment was given for at least 3 months after the procedure, and then, the decision was made according to the patient's CHA_2_DS_2_‐VASc score.^11^

### 2.2 Follow‐up

Patients were monitored post‐AF ablation at 1, 3, 6, and 12 months, followed by yearly visits. Follow‐ups included symptom assessment, physical exams, 12‐lead ECGs, and 24‐h Holter monitoring. If they had symptoms suggestive of ATa recurrence or procedure‐related complications, they were evaluated earlier. The presence of any ATa attack >30 s after ablation was considered a recurrence. Early recurrences were within the first 3 months (blanking period), while recurrences after 3 months were considered late.^11^ After AF ablation, all patients were given class IC or class III antiarrhythmic drugs for 3 months. After the ablation, the choice of antiarrhythmic drugs was left to the discretion of the physician. If re‐ablation was performed during the blanking period, the patient was considered as having early recurrence within 90 days and also having those at day 91.^12^

### 2.3 Statistical analysis

Categorical variables were given as numbers (percentages). “Shapiro‐Wilk” test was used to evaluate the distributions of continuous variables. Continuous variables with normal distribution were given as “mean ± SD,” and those without normal distribution were given as “median (interquartile ranges).” Differences between categorical variables were evaluated with the “chi‐squared test,” and differences between continuous variables were evaluated with the “Student *t*‐test” and “Mann–Whitney *U*‐test” for normally and non‐normally distributed ones, respectively. Using the receiver operating characteristic (ROC) analysis, the best cut‐off values of continuous variables that were statistically different between groups were determined. These cut‐off values were then used to create a risk score. Multiple Cox regression analysis was used to evaluate the effects of variables with significant differences between groups, and hazard ratio (HR) and 95% confidence interval (CI) were calculated. Regression coefficient (*β*) values of statistically significant variables were obtained from this analysis, and they were used to develop a risk score. Briefly, the *β* value of each variable was divided by the *β* value of the smallest, and the resulting value was rounded to the nearest integer. As a result, it was determined how many points the variables would receive.

We developed the APCEL risk score to estimate the risk of late recurrence. To test the ability of the APCEL score to predict late ATa recurrence, we used ROC analysis and calculated the area under the curve (AUC) value. We also calculated the AUCs of the previously defined risk scores and compared the AUC values of all risk scores. Since there were no patients >75 years of age in our study population, we did not include risk scores in which age >75 was a predictor.

We calculated the risk of developing late recurrence corresponding to each APCEL risk score and divided the patients into three groups according to the APCEL risk score: group 1 (score <2), group 2 (score: 2–3), and group 3 (score >3). We created Kaplan–Meier curves that show cumulative survival without late recurrence in each APCEL risk group and used the log‐rank test to determine differences between groups. Statistical analyses were performed using a software program (MedCalc® statistical software (Version 22.001)).

Time‐dependent ROC analysis was performed to evaluate the performance of the scores at specific time points (6, 12, 24, and 36 months) using survival data. The analysis employed the marginal weighting method, which utilizes the Kaplan–Meier estimator for the censoring distribution. Time‐dependent ROC curves and AUC ‐ values were calculated, and CIs were estimated using the influence function (IID) method. All analyses were implemented using the timeROC package in R.

## 3 RESULTS

### 3.1 Baseline characteristics

The age of the patients was 58.0 (50.0–63.0) years and 107 patients (51.9%) were male. Persistent AF was detected in 49 patients (23.8%) and AF duration before ablation was 20.0 (12.0–28.0) months. During the follow‐up period of 40.0 (21.0–57.0) months, early recurrence developed in 32 patients (15.5%), and late recurrence developed in 67 patients (32.5%). The frequencies of chronic obstructive pulmonary disease (COPD) (14.9% vs. 4.3%), persistent AF (38.8% vs. 17.3%), and early recurrence (35.8% vs. 5.8%) were significantly higher in those who developed late recurrence than in those who did not (*p* = .017, .001, and <.001, respectively). AF duration before ablation [26.0 (20.0–36.0) vs. 18.0 (9.0–24.0) months], left ventricle (LV) mass index [98.1 (85.9–115.5) vs. 93.0 (80.3–103.9) g/m^2^], LAVI [36.0 (30.0–44.0) vs. 28.0 (23.0–36.0) mL/m^2^], and systolic pulmonary artery pressure (SPAP) [27.0 (20.0–32.0) vs. 25.0 (20.0–28.0) mmHg] were significantly higher in those who developed late recurrence than in those who did not (*p* < .001, .017, <.001, and 0.019, respectively). Other baseline clinical features, medications, laboratory values, and other echocardiographic parameters were similar between the groups. The baseline clinical and laboratory features of the groups are presented in Table 1.

**TABLE 1:** Baseline characteristics of the study population (*n* = 206).

Parameters	Late recurrence (−) (*n* = 139)	Late recurrence (+) (*n* = 67)	*p*
Age (years)	59.0 (50.0–64.0)	58.0 (52.0–63.0)	.902
Sex (female) (*n*, %)	61 (43.9)	38 (56.7)	.084
BMI (kg/m^2^)	28.0 (25.7–32.1)	29.0 (26.8–32.7)	.131
Hypertension (*n*, %)	68 (48.9)	38 (56.7)	.294
Diabetes mellitus (*n*, %)	34 (24.5)	17 (25.4)	1.000
CAD history (*n*, %)	14 (10.1)	5 (7.5)	.727
HF with reduced EF (*n*, %)	4 (2.9)	6 (9.0)	.057
CVA/TIA (*n*, %)	10 (7.2)	5 (7.5)	.945
COPD (*n*, %)	6 (4.3)	10 (14.9)	.017
OSAS (*n*, %)	12 (8.6)	8 (11.9)	.617
Current smoking (*n*, %)	22 (15.8)	10 (14.9)	1.000
Alcohol consumption (*n*, %)	2 (1.4)	2 (3.0)	.451
Beta blocker (*n*, %)	98 (70.5)	52 (77.6)	.364
Amiodarone (*n*, %)	44 (31.7)	29 (43.3)	.139
Propafenone (*n*, %)	43 (30.9)	19 (28.4)	.829
Sotalol (*n*, %)	4 (2.9)	1 (1.5)	.545
Common trunk PV (*n*, %)	54 (38.8)	26 (38.8)	1.000
Accessory PV (*n*, %)	20 (14.4)	10 (14.9)	1.000
AF duration (months) (*n*, %)	18.0 (9.0–24.0)	26.0 (20.0–36.0)	<.001
Persistent AF (*n*, %)	24 (17.3)	26 (38.8)	.001
Early recurrence (*n*, %)	8 (5.8)	24 (35.8)	<.001
Follow‐up duration (months)	38.0 (17.0–57.0)	43.0 (27.0–56.0)	.247
BBB (*n*, %)	4 (2.9)	2 (3.0)	.966
CHA_2_DS_2_‐VASc score	2.0 (1.0–2.0)	2.0 (1.0–3.0)	.161
Hemoglobin (g/dL)	14.4 (12.9–15.5)	13.7 (12.8–14.5)	.070
eGFR (mL/dk/1.73 m^2^)	84.0 (73.0–96.8)	79.7 (68.2–95.6)	.273
LV EF (%)	61.0 (58.0–64.4)	61.0 (58.0–64.0)	.594
LV mass index (g/m^2^)	93.0 (80.3–103.9)	98.1 (85.9–115.5)	.017
LAVI (mL/m^2^)	28.0 (23.0–36.0)	36.0 (30.0–44.0)	<.001
TAPSE (mm)	24.1 ± 4.1	23.1 ± 3.6	.080
SPAP (mmHg)	25.0 (20.0–28.0)	27.0 (20.0–32.0)	.019

*Note*: CHA2DS2‐VASC: Heart failure/left ventricular ejection fraction <40%, hypertension, history of stroke or systemic embolism, age ≥ 75 years, diabetes mellitus, vascular disease, age 65–74 years, female sex.

Abbreviations: AF, atrial fibrillation; BBB, bundle branch block; BMI, body mass index; CAD, coronary artery disease; COPD, chronic obstructive pulmonary disease; CVA, cerebrovascular accident; EF, ejection fraction; eGFR, estimated glomerular filtration rate; HF, heart failure; LA, left atrium; LAVI, left atrium volume index; LV, left ventricle; OSAS, obstructive sleep apnea syndrome; PV, pulmonary vein; SPAP, systolic pulmonary artery pressure; TAPSE, tricuspid annular plane systolic excursion; TIA, transient ischemic attack.

### 3.2 Procedural features and follow‐up

For PV isolation, CB was used in 136 patients (66.0%) and RF ablation was used in 70 patients (34.0%). There was no difference in PV anatomical features between patients who developed late recurrence and those who did not (Table 1). When we analyzed separately according to the AF ablation method, there was no difference in the development of late recurrence in those who underwent CB and RF. Late recurrence developed in 45 (33.1%) of 136 patients who underwent CB and in 22 (31.4%) of 70 patients who underwent the RF ablation (*p* = .933). Antiarrhythmic drugs were successfully discontinued in all patients at the end of the first 3 months after AF ablation.

The causes of early recurrence were as follows: AF in 18 patients, typical AFL in six patients, atypical AFL in three patients, supraventricular tachycardia in three patients, AF and atypical AFL in two patients. The causes of late recurrence were as follows: AF in 44 patients, typical AFL in six patients, atypical AFL in three patients, AF and typical AFL in seven patients, AF and atypical AFL in three patients, typical and atypical AFL in two patients, and supraventricular tachycardia in two patients. During the blanking period, 10 patients underwent re‐ablation due to intolerable ATa that was resistant to drug therapy or cardioversion. Re‐ablation was performed in 27 of 67 patients who developed late recurrence. The responsible mechanisms in patients undergoing re‐ablation are shown in Table 2.

**TABLE 2:** Follow‐up data and procedure‐related complications.

	*n* (%)
Responsible mechanisms in patients undergoing re‐ablation during the blanking period	10 (4.9)
PV reconnection	1 (0.5)
CTI‐dependent typical AFL	6 (2.9)
LA roof‐dependent atypical AFL	1 (0.5)
Typical AVNRT	1 (0.5)
AT originating from Crista Terminalis	1 (0.5)
Responsible mechanisms in patients undergoing re‐ablation due to late recurrence^a^	27 (13.1)^a^
PV reconnection	17 (8.2)
CTI‐dependent typical AFL	11 (5.3)
LA roof‐dependent atypical AFL	6 (2.9)
Mitral isthmus‐dependent atypical AFL	3 (1.5)
LA posterior wall‐dependent atypical AFL	1 (0.5)
Procedure‐related complications	
Pericardial effusion without hemodynamic disturbance	4 (1.9)
Cardiac tamponade	4 (1.9)
Phrenic nerve paralysis	3 (1.5)
Moderate pulmonary vein stenosis	1 (0.5)
CVA/TIA	1 (0.5)
Groin complications	5 (2.4)

Abbreviations: AF, atrial fibrillation; AFL, atrial flutter; AT, atrial tachycardia; AVNRT, atrioventricular nodal reentrant tachycardia; CTI, cavotricuspid isthmus; CVA, cerebrovascular accident; LA, left atrium; PV, pulmonary vein; TIA, transient ischemic attack.

^a^ Some patients had two mechanisms of arrhythmia.

Post‐ablation complications are given in Table 2. Hemodynamic status improved with pericardiocentesis in all patients who developed cardiac tamponade (*n* = 4). Other patients with pericardial effusion were followed conservatively (*n* = 4). Of the five patients who developed groin complications, three had groin hematomas and two had arteriovenous fistulas. While patients with groin hematomas were followed conservatively, patients who developed arteriovenous fistulas underwent surgical repair.

### 3.3 Predictors of late recurrence

To find predictors of late ATa recurrence, we included seven variables that had a statistically significant difference between the groups in the multiple analysis (AF duration, persistent AF, early recurrence, COPD, LAVI, LV mass index, and SPAP). To develop a risk score, we performed an ROC analysis and determined cut‐off values for AF duration, LAVI, LV mass index, and SPAP. AF duration >19 months (AUC: 0.721, 95% CI: 0.655–0.781, *p* < .001), LAVI >31 mL/m^2^ (AUC: 0.690; 95% CI: 0.622–0.752, *p* < .001), LV mass index >111 g/m^2^ (AUC: 0.602, 95% CI: 0.532–0.670, *p* = .017), and SPAP >25 mmHg (AUC: 0.611, 95% CI: 0.531–0.668, *p* = .019) predicted late recurrence.

A multiple Cox regression analysis demonstrated that AF duration >19 months (HR: 3.18, 95% CI: 1.69–5.99), persistent AF (HR: 2.10, 95% CI: 1.08–4.07), COPD (HR: 2.66, 95% CI: 1.31–5.38), early recurrence (HR: 6.47, 95% CI: 3.53–11.86), and LAVI >31 mL/m^2^ (HR: 1.92, 95% CI: 1.02–3.59) were independent predictors for late recurrence (*p* < .001, .027, .006, <.001 and .041, respectively) (Table 3).

**TABLE 3:** Multiple Cox regression analysis results for prediction of late ATa recurrence after AF ablation.

	Beta	HR	(95% CI)	*p*
AF duration >19 months	1.15	3.18	1.69–5.99	<.001
Persistent AF	0.74	2.10	1.08–4.07	.027
COPD	0.97	2.66	1.31–5.38	.006
Early recurrence	1.86	6.47	3.53–11.86	<.001
LAVI >31 mL/m^2^	0.65	1.92	1.02–3.59	.041
LV mass index >111 g/m^2^	0.56	1.75	0.98–3.12	.057
SPAP >25 mmHg	0.42	1.53	0.86–2.703	.143

Abbreviations: AF, atrial fibrillation; ATa, atrial tachyarrhythmia; CI, confidence interval; COPD, chronic obstructive pulmonary disease; HR, hazard ratio; LAVI, left atrium volume index; LV, left ventricle; SPAP, systolic pulmonary artery pressure.

### 3.4 Risk scoring systems for late recurrence prediction

**FIGURE 2:**
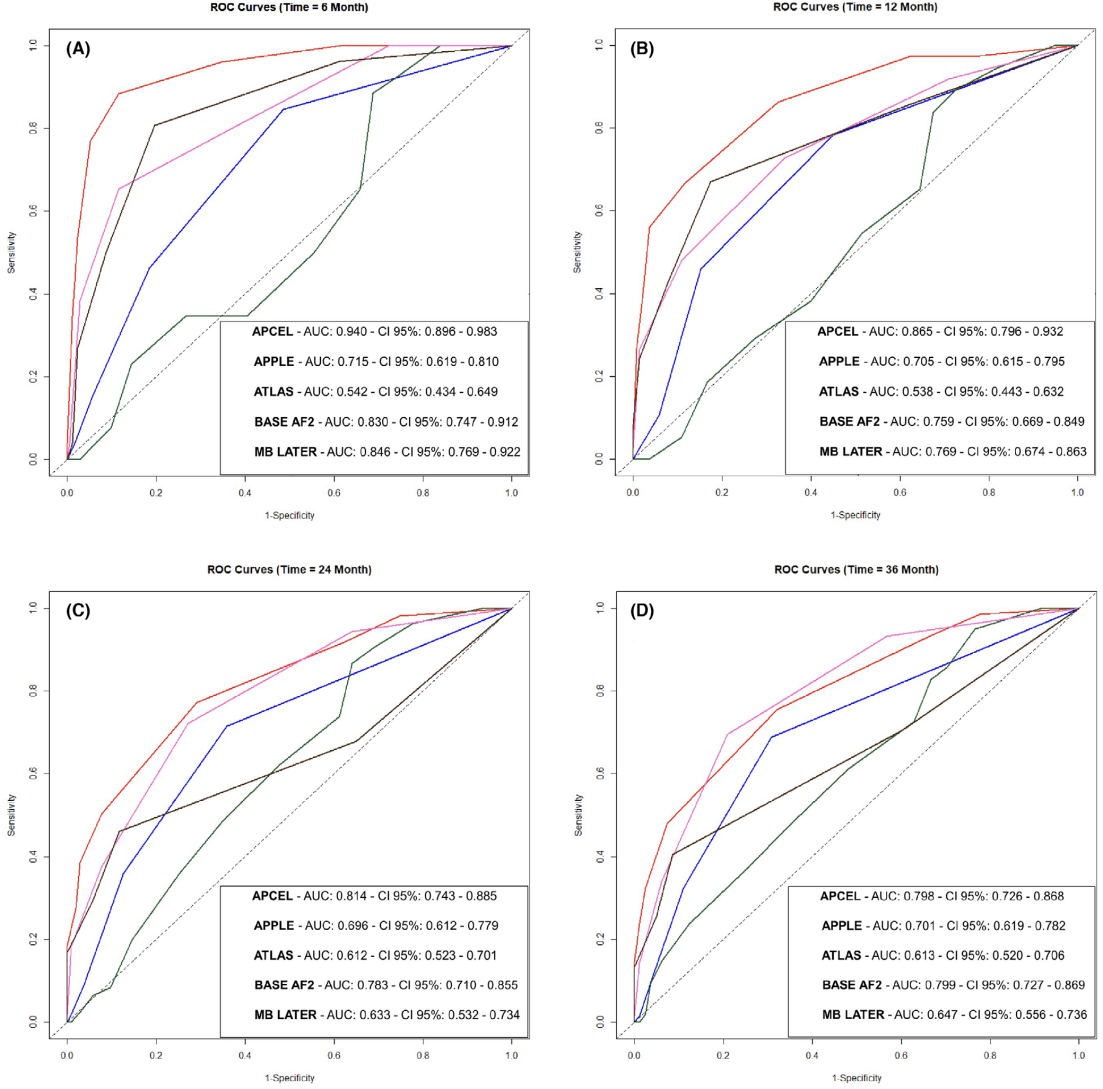
Time‐dependent ROC curves to evaluate late recurrence prediction performances of the scores at 6, 12, 24, and 36 months. ROC, receiver operating characteristic.

Using the five independent variables mentioned above, we developed the APCEL scoring system to predict late recurrence after AF ablation. According to the regression coefficients (*β*) of these variables, early recurrence received ‐three points, AF duration received two points, and others received one point (Table 4). We performed time‐dependent ROC analysis and calculated AUC values for the APCEL score at 6, 12, 24, and 36 months (Figure 2). The AUC for the APCEL score in predicting late recurrence was 0.940 (95% CI: 0.896–0.983) at 6 months, 0.865 (95% CI: 0.796–0.932) at 12 months, 0.814 (95% CI: 0.743–0.885) at 24 months, and 0.798 (95% CI: 0.726–0.868) at 36 months.

**TABLE 4:** APCEL score.

Variables		Score
A	AF duration >19 months	2
P	Persistent AF	1
C	COPD	1
E	Early recurrence	3
L	LAVI ≥ 31 mL/m^2^	1
Maximum points		8

Abbreviations: AF, atrial fibrillation; COPD, chronic obstructive pulmonary disease; LAVI, left atrium volume index.

In terms of predicting late recurrence, the APCEL score was superior to the ATLAS score at 6, 12, 24, and 36 months (*p* < .001, <.001, <.001, and =.002, respectively), the APPLE score at 6, 12, and 24 months (*p* < .001, =.005, and =.005, respectively), the MB LATER risk score at 6 and 36 months (*p* = .036 and =.009, respectively), and the BASE AF2 score at 6 months (*p* = .020). A comparison of the p values of the scoring systems is given in Table 5.

**TABLE 5:** Comparison of *p*‐values of scoring systems in the study.

	6th month	12th month	24th month	36th month
APCEL vs. APPLE	<.001	.005	.005	.079
APCEL vs. ATLAS	<.001	<.001	<.001	.002
APCEL vs. BASE AF2	.020	.068	.068	.983
APCEL vs. MB LATER	.036	.108	.108	.009
APPLE vs. ATLAS	.018	.011	.011	.165
APPLE vs. BASE AF2	.073	.404	.404	.076
APPLE vs. MB LATER	.035	.337	.337	.381
ATLAS vs. BASE AF2	<.001	<.001	<.001	.002
ATLAS vs. MB LATER	<.001	<.001	<.001	.614
BASE AF2 vs. MB LATER	.776	.884	.884	<.001

### 3.5 Predictive ability of APCEL score for late recurrence

**FIGURE 3:**
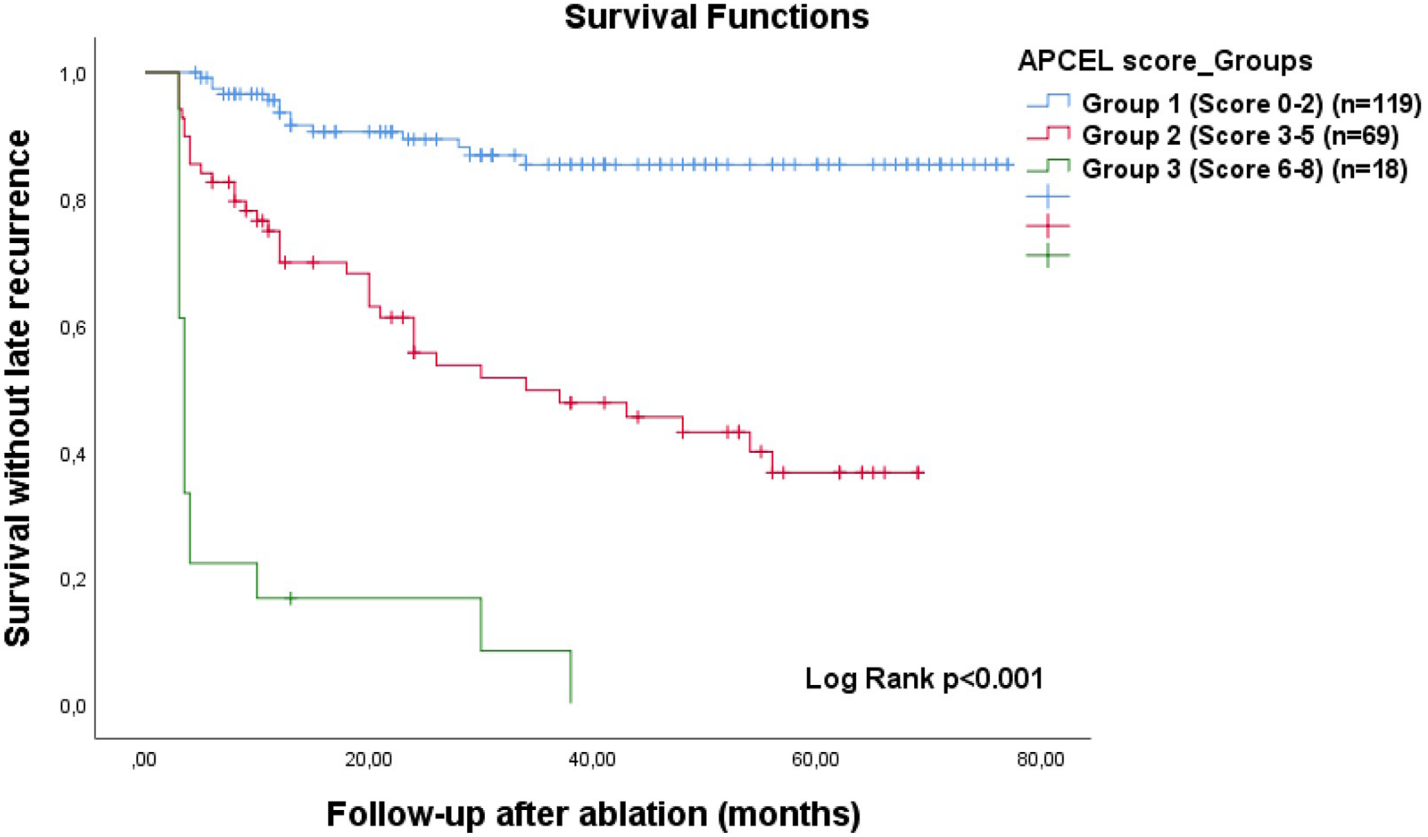
Kaplan–Meier curves showing late recurrence‐free survival after AF ablation with APCEL risk groups (Group 1: Score <3, Group 2: Score 3–5, Group 3: Score >5). AF, atrial fibrillation.

The median APCEL score was significantly higher in patients with late recurrence [3.0 (3.0–6.0)] than in those without [2.0 (0–2.0)] (*p* < .001). In general, the risk of late recurrence increased in proportion to the patients' APCEL scores. The risk of late ATa recurrence was 2.4% for those with an APCEL score of 0 (*n* = 42), 11.1% for those with 1 (*n* = 27), 20.0% for those with 2 (*n* = 50), 46.5% for those with 3 (*n* = 43), 66.7% for those with 4 (*n* = 15), 54.5% for those with 5 (*n* = 11), 100% for those with 6 (*n* = 7), 90.0% for those with 7 (*n* = 10), and 100% for those with 8 (*n* = 1). When the APCEL score was divided into three groups as <3 (group 1), 3–5 (group 2), and >5 (group 3), a Kaplan–Meier curve showed that late recurrence‐free survival was significantly different between APCEL groups 1, 2, and 3 (Log Rank *p* < .001) (Figure 3).

## 4 DISCUSSION

We found some predictors for the development of late recurrence in prolonged follow‐up after AF catheter ablation and defined the APCEL risk score. The main findings of our study were as follows: (1) During the follow‐up period, 15.5% of the patients developed early recurrence and 32.5% developed late recurrence. (2) Pre‐ablation AF duration >19 months, persistent AF, early recurrence, COPD, and LAVI >31 mL/m^2^ were independent predictors for the development of late recurrence. (3) The APCEL score, consisting of the combination of these markers (Early recurrence: 3 points, AF duration >19 months: 2 points, others: 1 point), had a good predictive ability for late recurrence at 6, 12, 24, and 36 months. (4) In terms of prediction of late recurrence, the APCEL score had better performance than the ATLAS and APPLE scores. In addition, the APCEL score was superior to the MB LATER score at 6 and 36 months and was superior to BASE AF2 score at 6 months. (5) As the APCEL score increased, the probability of late recurrence increased. When the patients were divided into three groups as group 1 (score <3), group 2 (score 3–5), and group 3 (score >5) according to APCEL risk scores, the risk of recurrence gradually increased from group 1 to 3.

Our study had some important strengths. In our study, the follow‐up period after AF ablation was longer (median 40 months) than most studies in which similar risk scores were developed^3^, ^5^, ^6^ and the AF duration before the procedure was longer (average 20 months) than in similar studies.^5^ We considered the development of early recurrence and persistent AF, which have previously been shown to be important predictors of late recurrence, to develop a new risk score. We used LAVI, which is a more realistic indicator of LA size, instead of LA anteroposterior diameter. We included patients who underwent PV isolation with both CB and RF, so we had the opportunity to evaluate the determinants of recurrence in patients who underwent ablation with both methods. Finally, we performed PV isolation during the first procedure in all patients and did not create empirical ablation lines. Thus, we eliminated the long‐term ATa‐producing effect of empirical ablation lines.

The time interval between AF diagnosis and PV isolation is crucial for predicting future recurrence.^1^, ^2^ Prolonged duration leads to structural and electrical atrial remodeling, making ablation less effective.^13^, ^14^, ^15^ Studies have shown that long‐term outcomes are better when catheter ablation is performed within the first year of AF diagnosis, and the probability of recurrence increases if the AF time before the procedure is prolonged.^14^, ^15^, ^16^, ^17^ AF duration was also one of the recurrence markers in the BASE‐AF2 score.^6^ We found AF duration >19 months before ablation to be an important predictor of late recurrence (2 points on APCEL score). The results of our study once again emphasize the importance of performing the procedure without delay to reduce the risk of recurrence in patients who are considered for catheter ablation for AF.

Other important parameters affecting the development of recurrence after AF ablation are AF type and LA size. We found the presence of persistent AF and LAVI >31 mL/m^2^ to be independent determinants of late recurrence. Studies have shown that the risk of recurrence in patients with persistent AF is higher than in those with paroxysmal AF.^18^, ^19^ The fact that atrial remodeling is more common due to both the longer duration of AF and LA dilation and the presence of more non‐PV triggers may explain the higher recurrence rate in patients with persistent AF.^20^, ^21^, ^22^ LA dilatation is indicative of atrial remodeling, and it is correlated with AF progression and the presence of atrial fibrosis.^23^ LA expansion is asymmetrical, occurring mainly in the mediolateral and superoinferior axes. For this reason, LA volume is a more realistic indicator of LA size than anteroposterior diameter measurement. In a meta‐analysis, LAVI was shown to be a risk factor for recurrence after catheter ablation.^8^

Gas exchange abnormalities like hypoxia and hypercapnia from COPD lead to pulmonary hypertension, resulting in right ventricular hypertrophy and diastolic dysfunction.^24^ Chronic hypoxia induces systemic inflammation and oxidative stress, promoting pro‐fibrotic changes in atrial tissue.^25^ COPD is identified as a predictor of recurrence in some risk scores after AF ablation.^5^, ^26^ Our study confirms COPD as an independent predictor of late recurrence post‐AF ablation.

Early recurrence of atrial fibrillation (AF) can manifest within days to weeks following catheter ablation, attributed to factors such as myocardial necrosis, oxidative stress, local and systemic inflammation, and subsequent tissue and nerve repair following ablation‐induced injury.^2^, ^27^ These mechanisms also play a crucial role in the emergence of late recurrence. In many studies and risk scoring, the presence of early recurrence is a predictor for the development of late recurrence.^6^, ^7^, ^28^, ^29^, ^30^ Consequently, we have integrated early recurrence as the most important independent predictor of late recurrence into our risk scoring system. This scoring framework is designed to identify patients who may require intensified surveillance for late recurrence or necessitate more rigorous management of reversible risk factors to mitigate the risk of recurrence. While some existing risk scores that exclude early recurrence can identify patients at high risk for late recurrence prior to the procedure,^3^, ^4^, ^5^ it is important to note that symptomatic AF patients often experience significant benefits from catheter ablation, regardless of their risk scores. Therefore, reliance solely on these scores may not be ideal for selecting candidates for ablation.

The APCEL risk score had very good performance in predicting the development of late recurrence at 6‐month follow‐up and was superior to other risk scores examined. While the APCEL score was generally superior to other risk scores in predicting late recurrence at follow‐up after 6 months, it was similar to the BASE AF2 risk score in terms of predicting late recurrence at follow‐up after 6 months.^6^ Early recurrence and AF duration are important variables in both APCEL and BASE AF2 risk scores. This may explain the similarity of both risk scores in terms of predicting late recurrence at follow‐up after 6 months.

The APCEL score calculated at the end of the blanking period can be used to determine the risk of recurrence in extended follow‐up. Patients with a high APCEL risk score can be monitored more closely as they may be more prone to the development of late recurrence. Again, modifiable risk factors such as hypertension, diabetes, and obstructive sleep apnea syndrome can be controlled more tightly to prevent further increases in the risk of recurrence in such patients.

### 4.1 Limitations

Limitations of our study include its single‐center retrospective design and the use of 24‐h Holter monitoring instead of continuous monitoring, potentially missing some asymptomatic recurrences. The small sample size of the study and the lack of a validation cohort are other important limitations. In our study, there was no data on AF burden, which is an important predictor for clinical outcomes after AF ablation, and there was no data on patient outcomes such as quality of life. The APCEL score's reliance on early recurrence limits its utility in predicting late recurrence risk pre‐ablation. Additionally, the absence of patients over 75 years old restricts the generalizability of our findings to this age group.

## 5 CONCLUSIONS

In patients undergoing AF catheter ablation, the APCEL score at the end of the blanking period predicts recurrence in prolonged follow‐up. Close monitoring of high‐risk patients can aid in detecting late recurrence, and controlling modifiable risk factors is crucial in such patients. To enable routine clinical use, further validation with a larger sample size in diverse patient groups, including those over 75, is needed.

## AUTHOR CONTRIBUTIONS

Conceptualization: Taner Ulus, Ertuğrul Çolak; Methodology: Taner Ulus; Formal analysis and investigation: Taner Ulus and Ahmet Şekip Ahmadi; writing—original draft preparation: Taner Ulus, Ahmet Şekip Ahmadi; writing—review and editing: Taner Ulus, Ahmet Şekip Ahmadi, Ertuğrul Çolak; Supervision: Taner Ulus, Ahmet Şekip Ahmadi, Ertuğrul Çolak.

## CONFLICT OF INTEREST STATEMENT

Authors declare no conflict of interests for this article.

## ETHICS STATEMENT

The study was approved by the local ethics committee.
